# Regulation of GABA_A_ and 5-HT Receptors Involved in Anxiolytic Mechanisms of Jujube Seed: A System Biology Study Assisted by UPLC-Q-TOF/MS and RT-qPCR Method

**DOI:** 10.3389/fphar.2020.01320

**Published:** 2020-10-15

**Authors:** Liang Chen, Xue Zhang, Chun Hu, Yi Zhang, Lu Zhang, Juntao Kan, Bo Li, Jun Du

**Affiliations:** ^1^Nutrilite Health Institute, Amway (China) R&D Center, Shanghai, China; ^2^Nutrilite Health Institute, Amway Innovation and Science, Buena Park, CA, United States

**Keywords:** anxiety, jujube seed, anxiolytic mechanism, system biology, 5-HT receptors, GABA_A_ receptors

## Abstract

The increase of the prevalence of anxiety greatly impacts the quality of life in China and globally. As the most popular traditional Chinese medicinal ingredient for nourishing health and tranquilizing mind, Jujube seed (*Ziziphus jujuba* Mill., Rhamnaceae) (SZJ) has been proved to exert anxiolytic effects in previous reports. In this study, a system biology method assisted by UPLC-Q-TOF/MS and RT-qPCR was developed to systematically demonstrate the anxiolytic mechanisms of SZJ. A total of 35 phytochemicals were identified from SZJ extract (*Ziziphus jujuba* Mill. var. spinosa [Bunge] Hu ex H.F. Chow), which interact with 71 anxiolytic targets. Protein-protein interaction, genes cluster, Gene Ontology, and Kyoto Encyclopedia of Genes and Genomes (KEGG) pathways analysis were subsequently conducted, and results demonstrated that regulation of serotonergic and GABAergic synapse pathways were dominantly involved in the anxiolytic mechanisms of SZJ extract. The effects of SZJ extract on mRNA expressions of multiple GABA_A_ (gamma-aminobutyric acid type A) and 5-HT (serotonin) receptors subtypes were further validated in human neuroblastoma SH-SY5Y cells using RT-qPCR. Results showed that SZJ extract (250 μg/mL) significantly up-regulated the mRNA level of GABRA1 and GABRA3 as well as HTR1A, HTR2A, and HTR2B in non-H_2_O_2_ treated SH-SY5Y cells. However, it exerted an inhibitive effect on the overexpressed mRNA of GABRA1, GABRA2, HTR1A, and HTR2A in H_2_O_2_ treated SH-SY5Y cells. Taken together, our findings suggest that anxiolytic mechanisms of SZJ mostly involve the regulation of GABAergic and serotonergic synapse pathways, especially a two-way modulation of GABRA1, HTR1A, and HTR2A. Our current results provide potential direction for future investigation of SZJ as an anxiolytic agent.

## Introduction

Anxiety is characterized as excessive and persistent worry about the future, which in turn can impact one’s ability to carry out activities of daily living. Anxiety can be divided into generalized anxiety disorder, panic disorder, obsessive-compulsive disorder, social anxiety disorder, and posttraumatic stress disorder (Möhler, 2012; Cohen et al., 2015). Physiological anxiety symptoms include pounding heart, difficulty breathing, upset stomach, muscle tension, sweating, and feeling faint or shaky (Teychenne et al., 2015). The global prevalence of anxiety is estimated at 16.6% across the life span, and it becomes a burden of healthcare and quality of life (Somers et al., 2006); therefore, it is important to develop effective and safe solutions for treatment (Starcevic, 2006). Tricyclic antidepressants, serotonin-specific reuptake inhibitors, and benzodiazepines have been developed to mitigate anxiety. While effective, these classes of drugs come with many side effects, such as insomnia, sexual dysfunction, suicidal ideation, and/or drug-dependency (Lakhan and Vieira, 2010). Therefore, the use of complementary and alternative medicines to improve anxiety has received increased attention. Traditional medicinal materials, such as Jujube seed (*Ziziphus jujuba* Mill., Rhamnaceae) (SZJ) (Huang et al., 2008; Wang Y. et al., 2008), saffron (*Crocus sativus* L.) (Lopresti et al., 2018; Milajerdi et al., 2018), valerian root (*Valeriana officinalis* L.), and passion flower (*Passiflora incarnata* L.) (Müller et al., 2003; Cass, 2004; Benke et al., 2009) have shown anti-anxiety benefits.

SZJ, also known as *Suanzaoren* in Chinese, was first recorded in *Shennong Bencao Jing*, the earliest classic treatise of Chinese Materia Medica. SZJ has a long history of use in China as a vital food and/or medicine that traditionally is considered to sustain human health by calming the mind and improving the quality of sleep. In recent years, accumulated evidences have shown that SZJ and/or its preparations exert positive outcome on insomnia (Jiang et al., 2007; Chen et al., 2011; Ni et al., 2015; Shergis et al., 2017; Xiao et al., 2018), anxiety (Peng et al., 2000; Liu et al., 2015), and depression (Liu et al., 2012; Liang et al., 2016), mainly through regulating GABAergic (Chen et al., 2007; Chen, 2008; Shergis et al., 2017) and serotoninergic systems (Wang L.-E. et al., 2008; Wang et al., 2010; Liu et al., 2015). Jujubosides (e.g., jujuboside A, B), C-glycoside flavonoid (e.g., spinosin), and pentacyclic triterpenic acid (e.g., betulinic acid) have been identified from SZJ (Liu et al., 2007; Zhang et al., 2008; Lee et al., 2016) as the potential active phytochemicals contributing to these healthy benefits.

Although previous studies have shown promising anxiolytic effects of SZJ, the underlying mechanism has not been systematically and comprehensively investigated. In our current work, we developed an integrated strategy of system biology assisted by ultra-performance liquid chromatography quadrupole-time of flight mass spectrometer (UPLC-Q-TOF/MS) and real-time quantitative reverse transcription polymerase chain reaction (RT-qPCR) to uncover the active phytochemicals and anxiolytic mechanism of SZJ extract. This approach provides a modern and practical way to study complicated chemical systems with multiple pathways and connected targets, which is otherwise a difficult challenge in mechanistic research of traditional Chinese medicine ingredients.

## Material and Method

### Phytochemical Analysis of SZJ Extract Using Ultra-Performance Liquid Chromatography Quadrupole-Time of Flight Mass Spectrometer (UPLC-Q-TOF/MS)

#### Characteristics of the SZJ Extract

Commercial SZJ extract (batch number HS-180651) was purchased from Honsea Sunshine (Guangzhou, China). Dried seed of *Ziziphus jujuba* Mill. var. spinosa (Bunge) Hu ex H.F. Chow was used for SZJ extract production in Guangzhou, China. SZJ was extracted by supercritical fluid CO_2_ to remove lipid fraction, and the residue was further extracted by 50% ethanol, followed by vacuum concentration and vacuum drying. The final extraction ratio is 5:1, and total content of jujuboside A and B was quantitatively detected as 0.20% using HPLC method. A voucher of the batch used has been deposited at -16° refrigerator, sampler chamber of Amway (China) R&D Center (Shanghai, China).

#### Sample Preparation

Thirty mg of SZJ extract powder was precisely weighted and then transferred to a centrifugal tube, with 1.5 mL of methanol (Mass Pure Grade from MERCK), ultrasonicated for 30 min (KQ-300DB, 300W,40kHZ) at ambient temperature, followed by centrifuge (12000 rpm, 5 min, SIGMA 3K15, SIGMA). The obtained supernatant is filtered through 0.22 μm filter member prior to UPLC-Q-TOF/MS analysis.

#### UPLC-Q-TOF/MS Conditions

Chemical profiling was performed on an Agilent 1290 UPLC system (Agilent Technologies, Palo Alto, USA) coupled with Sciex TripleTOF 4600^®^ quadrupole-time of flight mass spectrometer (AB Sciex, Darmstadt, Germany) equipped with a DuoSpray source (electrospray ionization, ESI). Agilent SB C18 column (2.1×100 mm i.d., 1.8 μm; Agilent) was used for components separation. The mobile phase consisted of water containing 0.1% formic acid (A) and acetonitrile (B). The following gradient condition was used: 0–2.0 min, 5%–5% B; 2.0–10.0 min, 5%–30% B; 10.0–15.0 min 30%–50% B; 15.0–25.0 min, 50%–95% B; 25.0–27.0 min, 95%–95% B, with the flow rate of 0.3 mL/min. The injection volume was 1 μL, while column oven temperatures was set at 25°C. The mass spectrometer was operated in full-scan TOF-MS at m/z 100–1500 and information-dependent acquisition (IDA) MS/MS modes, with both positive and negative ion modes. The collision energy was -40 ± 20 eV, ion source gas 1 and 2 were set 50 psi, curtain gas was 35 psi. The temperature and ion spray voltage floating were 500°C and 5000/-4500 V, respectively.

#### Data Analysis

Data recording and processing was performed by Analyst software (Version 1.6, AB Sciex, USA). The compounds were tentatively characterized based on their retention time, mass accuracy of precursor ions, MS/MS spectra, and fragmentation pathways, referring to the SCIEX natural products HR-MS/MS Spectral Library, standard references, and previous literatures.

### System Biology Analysis of SZJ Extract With Anxiolytic Effects

#### Construction of Anxiety-Related Targets Database

A text mining of National Center for Biotechnology Information (NCBI) (https://www.ncbi.nlm.nih.gov/gene/), Integrative Pharmacology–based Research Platform of Traditional Chinese Medicine (TCMIP, http://www.tcmip.cn/TCMIP/index.php/Home/) (Xu et al., 2019), and Comparative Toxico-genomics Database (CTD, http://www.ctdbase.org/) (Davis et al., 2019) was conducted to retrieve anxiety-related targets with the keywords “anxiety.” TCMIP integrates the diseases related genes data of Therapeutic Targets Database (https://db.idrblab.org/ttd/), Human Phenotype Ontology database (HPO, https://hpo.jax.org/app/), and DisGeNET database (https://www.disgenet.org/). The search results of targets from NCBI, TCMIP, and CTD were filtered with “Homo sapiens,” and only the targets with direct evidence supported by CTD were selected. All acquired targets were combined and then mapped to UniProt (https://www.uniprot.org/) for normalization and removal of duplicate and erroneous targets (Liu J. et al., 2018). The remaining satisfactory targets constitute the anxiety-related gene targets database.

### Acquisition of Potential Targets Regarding Anxiolytic Benefits for Identified Phytochemicals

The targets of identified phytochemicals and their potential metabolisms were acquired from multiple databases. In addition to retrieving candidate targets from TCMIP and CTD platforms, PharmMapper Server (http://www.lilab-ecust.cn/pharmmapper/) (Wang et al., 2017) was employed to fish the potential targets for those phytochemicals of which no available candidate targets were found in TCMIP and CTD. Those targets were excluded if their reliable score was lower than 0.8 from TCMIP, interaction counts less than 5 from CTD, and/or their normalized fit score lower than 0.8 from PharmMapper. After removal of the duplicates, erroneous, and non-Homo sapiens targets, the rest were then mapped to an anxiety-related gene targets database to screen out the intersecting targets.

#### Protein-Protein-Interaction (PPI) and Clusters Analysis.

The selective target genes of SZJ extract were imported to STRING (Version 11.0, https://string-db.org/) (Szklarczyk et al., 2019) to obtain PPI results. The interaction score set as high confidence (>0.7). The STRING analysed results were then imported into Cytoscape (Version 3.6.1) (Su et al., 2014), and cluster analysis of target genes was conducted using a Molecular Complex Detection (MCODE) plug-in according to the method in the literature (Wang et al., 2020).

#### Enrichments Analysis Along With Network Construction.

The target genes contained in the MCODE enriched clusters were imported into the database for annotation, visualization, and integrated discovery (DAVID, version 6.8) (https://david.ncifcrf.gov/tools.jsp) (Huang et al., 2009) to conduct Gene Ontology (GO) terms enrichment including biological processes, cell component, and molecular function. ClueGo plug-in (Version 2.5.7) (Bindea et al., 2009) was further employed to analyze and demonstrate their participated biologic process and Kyoto Encyclopedia of Genes and Genomes (KEGG) pathways, respectively. The latest databases of GO-Biologic Process annotation EB1-Uniport and KEGG pathway were selected. Visual style was set as “groups,” minimum gene number, and percentage contained in a term set as 5 and 5%. Statistical method of two-sided hypergeometric test and Bonferroni step down p-value correction was used. Cut-off value of kappa score of GO term/pathway network connectivity was set as 0.5, and only term/pathway with p-value < 0.05 was shown.

### RT-qPCR Test

#### Samples Preparation

Gamma-amino butyric acid (GABA) was kindly provided by Toong Yeuan International Group (Shanghai, China). GABA was considered as positive control in this test. SZJ extract and GABA ingredients were dissolved in deionized water and diluted in culture solution before use.

#### Cell Culture

Human neuroblastoma SH-SY5Y cells were kindly provided by Stem Cell Bank, Chinese Academy of Sciences, and cultured in MEM/F12 (Gibco) supplemented with 10% (v/v) inactivated fetal bovine serum (Gibco), 1% Gluta-max (Gibco), 1% Sodium pyruvate (Gibco), 1% NEAA (Gibco), and 100 U/mL penicillin/streptomycin. The cells were maintained at 37° in 5% CO_2_ and 95% humidified air incubator for the indicated time. All experiments were carried out 24 h after cells were seeded.

#### Samples Concentration Determination

CellTiter-Glo assay was used to evaluate the available concentration of test samples according to the method described in literature (Fallahi-Sichani et al., 2013). The inhibition on SH-SY5Y cell viability of a series of concentrations of SZJ extract and GABA samples, from 3 mg/mL-0.46 μg/mL, were respectively evaluated. The nonlinear fitting curve of logarithm concentration response to cells viability was then simulated by GraphPad Prism 7.0 (GraphPad Software Inc., San Diego, CA, USA). The concentration of SZJ extract for further test referred to the correspondence value of 90% cells viability.

#### Samples Stimulation and Grouping

A total of 6 group samples were simultaneously tested: control group (deionized water), 250 μg/mL SZJ extract group, 100 μg/mL GABA group, 100 μM H_2_O_2_ group, 250 μg/mL SZJ extract with 100 μM H_2_O_2_ group, and 100 μg/mL GABA with 100 μM H_2_O_2_ group. Stimulation duration of all group samples was 48 h. Duplicates for each group were set as 3.

#### RNA Isolation and Reverse Transcription

RNA isolation and reverse transcription were conducted following the method reported in the literature (Fuchsova et al., 2016). Briefly, RNAprep Pure Cell/Bacteria kit (Tiangen, China) was used to extract total RNA according to the manufacturer’s instructions. NanoDrop One^C^ (ThermoFisher, USA) was used to determine RNA yield and purity by absorbance ratios A260/A280 and A260/A230. OD260/OD280 ratios of the RNA of all samples were in the range of 1.8–2.0. 2 μg of total RNA used to synthesize the first strand complementary DNA (cDNA) using high-capacity cDNA reverse transcription kit (ThermoFisher, USA) according to manufacturer’s directions. All reverse transcription products were 10-fold dilution.

#### Oligonucleotide Primers

PrimerBank was applied to search primers for the amplification of human GABRA1, GABRA2, GABRA3, HTR1A, HTR1B, HTR2A, HTR2B, and internal reference genes (GAPDH and ACTB). Nucleotide sequence of primers are listed in **Table 1**.

**Table 1:** Nucleotide sequence of the forward and reverse primers, the lengths of the PCR products.

Target mRNA bases	Primer sequences	T_m_ (°C)	PCR products (bp)
GABRA1	Forward, 5’ AGCCGTCATTACAAGATGAACTT 3’ Reverse, 5’ TGGTCTCAGGCGATTGTCATAA 3’	60 61.2	95
GABRA2	Forward, 5’ GCTGGCTAACATCCAAGAAGAT3’	60.1	92
	Reverse, 5’ GCCGATTATCGTAACCATCCAGA3’	61.9	
GABRA3	Forward, 5’ CAAGGGGAATCAAGACGACAA 3’ Reverse, 5’ CGTCCAGAAGACGATCCAAGAT 3’	60 61.5	145
HTR1A	Forward, 5’ ACCATTAGCAAGGATCATGGC 3’ Reverse, 5’ ATATGCGCCCATAGAGAACCA 3’	60.2 60.8	94
HTR1B	Forward, 5’ GGGTTCCTCAAGCCAACTTATC 3’ Reverse, 5’ GCCAATAGCATAACCAGCAGT 3’	60.6 60.8	115
HTR2A	Forward, 5’ TTAAGGAGGGGAGTTGCTTACT 3’ Reverse, 5’ TGCCAAGATCACTTACACACAAA 3’	55.1 54.1	156
HTR2B	Forward, 5’ TGATTTGCTGGTTGGATTGTTTG 3’ Reverse, 5’ ATGGATGCGGTTGAAAAGAGAA 3’	53.9 54.3	132
β-actin (ACTB)	Forward, 5’ CTTCGCGGGCGACGAT 3’ Reverse, 5’ CCACATAGGAATCCTTCTGACC 3’	65.1 63.1	104
GAPDH	Forward, 5’ GGAAGGTGAAGGTCGGAGTC 3’ Reverse, 5’ TGGAATTTGCCATGGGTGGA3’	64.9 65.5	166

#### Real-Time PCR (qPCR).

Levels of mRNA were quantified by conducting qPCR reactions with SsoAdvanced Universal SYBR Green Supermix (Bio-Rad, USA) according to the manufacturer’s directions. CFX equipped with software (Bio-Rad, USA) was used to perform measurements. PCR was in the 20 μL reaction system containing 0.5 μM primer, 10 μL Mix, 5 μL cDNA, and 3 μL RNase-free ddH_2_O, and the amplification and dissolution curve condition was shown in **Table 2**. qPCR amplification of ACTB (β-actin) and GAPDH (glyceraldehyde-3-phosphate dehydrogenase) transcription were used as the internal control to verify that equal amounts of RNA were used in each reaction. Fold expression was defined as the fold change relative to control cells.

**Table 2:** Condition of amplification and dissolution curve.

Stage	Temperature (°C)	Time	Number of cycles
Ding stage Cycling stage Melt curve stage	95 95 60 65–95°C 0.5°C increment	30s 10s 20s	1 40 1

#### Statistical Analysis

Bio-Rad CFX Manager software (Bio-Rad, USA) was employed to analyze raw expression data (CT values). For further statistical analysis we used values normalized to the normalization factor calculated as a geometric mean of the expression of two reference genes. All data were expressed as the mean ± SD, and a two-way ANOVA followed by Tukey test was applied for statistical analysis using GraphPad Prism 7.0.

## Results

### Phytochemicals Identification of SZJ Extract

**Figure 1:**
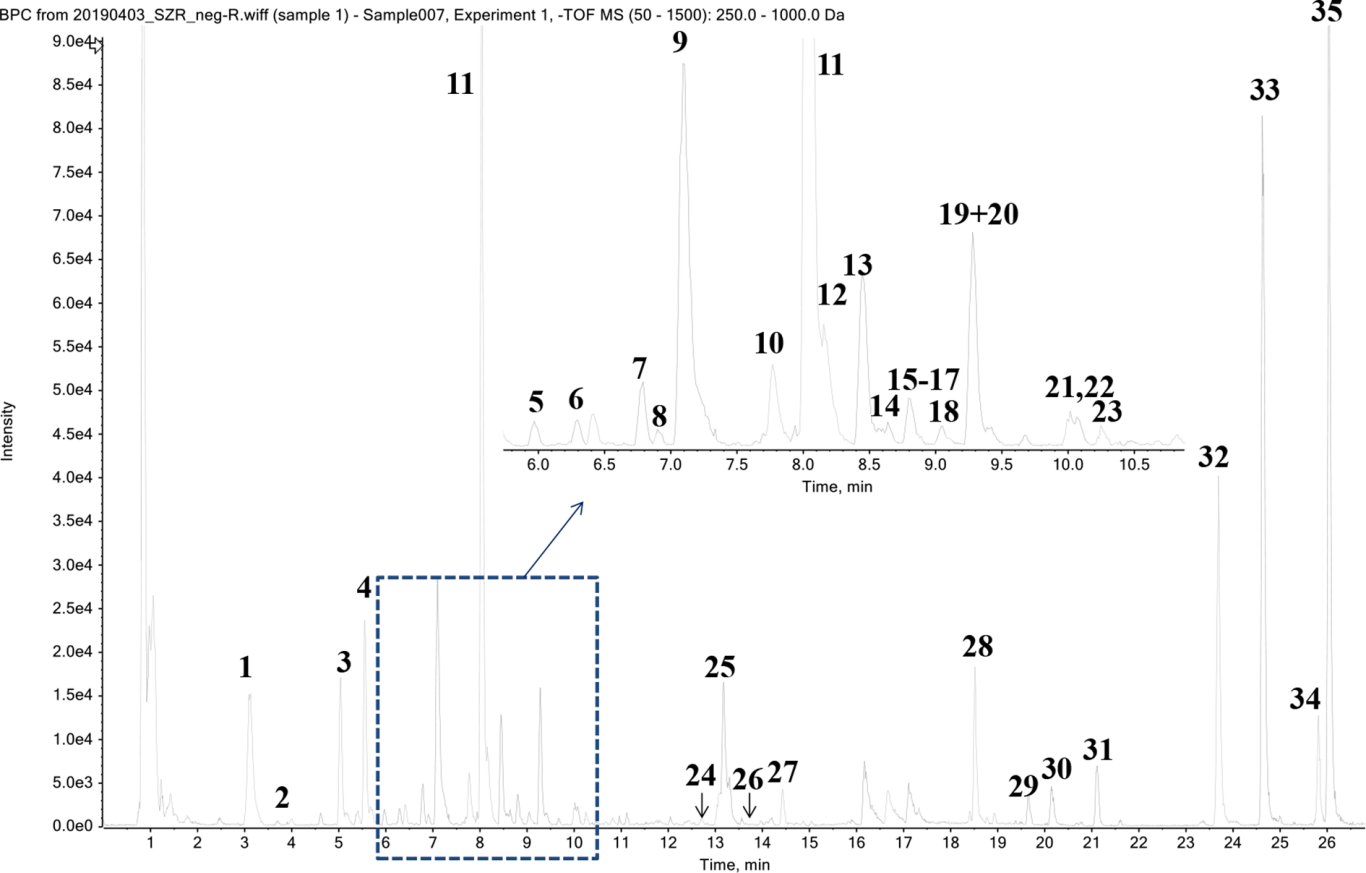
Chromatographic profile of SZJ extract using UPLC-Q-TOF/MS in negative ion mode.

Thirty-five phytochemicals were identified from SZJ by using the UPLC-Q-TOF/MS method. The MS chromatogram in negative ion model of SZJ extract is shown in **Figure 1**. The chemical information of identified phytochemicals is seen in **Table 3**. Among them, 11 phytochemicals were identified by comparison with standard references. The rest of the compounds were identified through comparison with literature data. These phytochemicals are mainly classified into four subcategories: (1) saponins including jujuboside A and jujuboside B; (2) flavones and their C-glycosides including catechin, epicatechin, vicenin-2, swertisin, nicotiflorin, isovitexin and its analogues, and spinosin and its analogues; (3) organic acids including triterpenic acid (e.g., alphitolic acid), fatty acid (e.g., linoleic acid), and glycosylated organic acids (pseudolaroside B and oleuropein); and (4) alkaloids including indoleacetic acid derivatives (e.g., N-glc-indoleacetic acid) and isoquinoline alkaloids (zizyphusine).

**Table 3:** Identified phytochemicals in SZJ extract.

No	RT (min)	Adducts	Measured *m/z*	Expected *m/z*	Mass error(ppm)	Formula	Molecular weight	Phytochemical name	MS/MS fragment ions	Reference
S1	3.11	[M-H]^-^	368.0999	368.0987	3.2	C_16_H_19_NO_9_	369.11	3S-N-glc-3-hydroxy-indoleacetic acid	368.0062,204.0059,176.0160,158.0085,143.9957,130.0195,	(Zhang F.-X. et al., 2016)
S2	3.70	[M-H]^-^	329.0885	329.0878	2.1	C_14_H_18_O_9_	330.29	Pseudolaroside B	166.9794,151.9582	(Zhang F.-X. et al., 2016)
S3	5.04	[M-H]^-^	352.1050	352.1038	3.4	C_16_H_19_NO_8_	353.11	N-glc-indoleaceticacid	352.0176,308.0360,188.0133,160.0243,146.0105,	(Zhang F.-X. et al., 2016)
S4	5.55	[M-H]^-^	352.1054	352.1038	4.6	C_16_H_19_NO_8_	353.11	isomer of N-glc-indoleaceticacid	352.0161,308.0324,188.0126,160.0231,146.0103,	(Zhang F.-X. et al., 2016)
S5	5.97	[M-H]^-^	289.0724	289.0718	2.2	C_15_H_14_O_6_	290.08	catechin	288.9944,245.0123,202.0026,173.8291,122.9996,	a
S6	6.29	[M-H]^-^	595.1694	595.1668	4.3	C_27_H_32_O_15_	596.17	5,7-dihydroxy-2-(4-hydroxyphenyl)6,8-bis[3,4,5-trihydroxy-6(hydroxymethyl)oxan-2-yl]-2,3dihydrochromen-4-one	595.0439,475.0168,385.0004,354.9931	(Khan et al., 2020)
S7	6.78	[M-H]^-^	593.1544	593.1512	5.4	C_27_H_30_O_15_	594.16	vicenin 2	593.0275,473.0027,382.9841,352.9774	a
S8	6.90	[M-H]^-^	289.0722	289.0718	1.5	C_15_H_14_O_6_	290.08	epicatechin	288.9950,245.0136,203.0092,186.9836,136.9752,108.9860,	a
S9	7.10	[M-H]^-^	340.1574	340.1554	5.8	C_20_H_23_NO_4_	341.16	zizyphusine	340.0703,325.0486,310.0285,251.9723,223.9819,195.9970,	(Zhang F.-X. et al., 2016)
S10	7.77	[M-H]^-^	593.1533	593.1512	3.5	C_27_H_30_O_15_	594.16	isovitexin-2″-O-glucopyranoside	593.0285,412.9906,310.9741,292.9673,	(Wang et al., 2009)
S11	8.04	[M-H]^-^	607.1698	607.1727	-4.8	C_21_H_36_O_20_	608.18	spinosin	607.0438,445.0129,427.0056,306.9819,	a
S12	8.22	[M-H]^-^	431.1003	431.0984	4.5	C_21_H_20_O_10_	432.11	isovitexin	412.9913,340.9805,310.9738,282.9840,239.0020,	(Niu et al., 2010)
S13	8.45	[M-H]^-^	445.115	445.114	2.2	C_22_H_22_O_10_	446.12	swertisin	445.0127,324.9879,296.9616,281.9768,	(Wang et al., 2009)
S14	8.63	[M-H]^-^	757.2028	757.1985	1.8	C_36_H_38_O_18_	758.21	6’’’-vanilloylspinosin	757.0502,427.0029,268.9916,208.9818,	(Zhang F.-X. et al., 2016)
S15	8.81	[M-H]^-^	593.1542	593.1512	5.1	C_27_H_30_O_15_	594.16	nicotiflorin	593.0301,284.9642,254.9591,	a
S16	8.82	[M-H]^-^	727.1921	727.188	5.7	C_35_H_36_O_17_	728.20	6’’’-para-hydroxylbenzoylspinosin	727.0437,427.0015,238.9858,178.9772,136.9742,	(Niu et al., 2010)
S17	8.89	[M-H]^-^	769.1986	769.1985	0.1	C_37_H_38_O_18_	770.21	isovitexin-2″-O-(6-feruloy)-gluc-opyranoside	593.0295,412.9894,292.9667,234.9919,	(Zhang F.-X. et al., 2016)
S18	9.08	[M-H]^-^	813.2329	813.2246	10	C_39_H_42_O_19_	814.23	6’’’-sinapoylspinosin	813.0779,607.0452,427.0041,325.0097	(Wang et al., 2009)
S19	9.27	[M-H]^-^	753.2087	753.2036	6.7	C_37_H_38_O_17_	754.21	6’’’-para-coumaroylspinosi	753.0614,607.0459,427.0065,306.9802,264.9971,	(Niu et al., 2010)
S20	9.28	[M-H]^-^	783.2183	783.2142	5.2	C_38_H_40_O_18_	784.22	6’’’-feruloyspinosin	783.0707,427.0056,234.9936,192.9904,	(Wang et al., 2009)
S21	9.92	[M-H]^-^	869.2945	869.2874	8.2	C_43_H_50_O_19_	870.29	6’’’-(-)-phaseoylspinosin	869.1331,839.1245,607.0473,427.0105,	(Zhang F.-X. et al., 2016)
S22	10.04	[M-H]^-^	539.1786	539.177	2.9	C_25_H_32_O_13_	540.51	oleuropein	539.0602,307.0033,275.0163	(Rached et al., 2019)
S23	10.26	[M-H]^-^	783.2172	783.2142	3.8	C_38_H_40_O_18_	784.22	6’’-O-feruloylspinosin	783.0707,607.0454,445.0149,160.9688	(Wang et al., 2009)
S24	12.68	[M+HCOO]^-^	1251.608	1251.602	4.8	C_58_H_94_O_26_	1206.60	jujuboside A	1244.5868,1207.3994,1075.3781,	a
S25	13.12	[M-H]^-^	329.2349	329.2333	4.7	C_18_H_34_O_5_	330.46	octadecenoic acid	329.1494,229.0779,211.0714,171.0473	
S26	13.47	[M+HCOO]^-^	1089.555	1089.549	5.9	C_52_H_84_O_21_	1044.55	jujuboside B	1043.3670,911.3407,749.3084,	a
S27	14.43	[M-H]^-^	329.2349	329.2333	4.7	C_18_H_34_O_5_	330.46	octadecenoic acid	329.1487,201.0517,171.0472	
S28	18.51	[M-H]^-^	485.3305	485.3272	6.7	C_30_H_46_O_5_	486.33	epiceanothic acid	485.2225,423.2285,	(Yang et al., 2013)
S29	19.65	[M-H]^-^	295.2296	295.2279	5.9	C_18_H_32_O_3_	296.24	octadecenoic acid methyl ester	295.1481,277.1445,195.0798,155.0569,	
S30	20.17	[M-H]^-^	471.3516	471.348	7.7	C_30_H_48_O_4_	472.36	alphitolic acid	471.2459	(Yang et al., 2013)
S31	21.11	[M-H]^-^	485.329	485.3272	3.6	C_30_H_46_O_5_	486.33	ceanothic acid	485.2209,423.2296,	(Yang et al., 2013)
S32	23.69	[M-H]^-^	455.3555	455.3531	5.3	C_30_H_48_O_3_	456.36	betulinic acid	455.2508	a
S33	24.64	[M-H]^-^	279.2345	279.233	5.5	C_18_H_32_O_2_	280.24	linoleic acid	279.1583	a
S34	25.82	[M-H]^-^	255.233	255.233	0.2	C_16_H_32_O_2_	256.24	palmitic acid	255.1609	a
S35	26.04	[M-H]^-^	281.2492	281.2486	2.1	C_18_H_34_O_2_	282.26	oleic acid	281.1739	a

a, identified by standard references.

### Potential Metabolic Products by Gut Microbes of C-Glycoside and Jujubosides Phytochemicals

Gut microbes are known to deglycosylize and cleavage ester bond of flavone C-glycosides and their derivates (Kim et al., 2015; Vollmer et al., 2018; Zheng et al., 2019). Similarly, jujuboside A is metabolized to jujubogenin in gastrointestine to exhibit effects on the expression and activation of gamma amino-butyric acid A (GABA_A_) receptors (Song et al., 2017). Based on the metabolic patterns reported in those literatures, we deduced the metabolic products by gut microbe of flavone C-glycosides and jujubosides of SZJ extracts. As a result, 11 metabolites of these flavone C-glycosides and jujubosides are concluded for further system biology analysis. To be specific, ferulic acid is metabolized from 6’’’-feruloyspinosin and 6’’-O-feruloylspinosin, para-coumaric acid is from 6’’’-para-coumaroylspinosin, phaseic acid is from 6’’’-(E)-phaseolspinosin, para-hydroxybenzoic acid is from 6’’’-para-hydroxyl-benzoylspinosin, sinapic acid is from 6’’’-sinapoylspinosin, vanillic acid is from 6’’’-vanilloyl-spinosin, kaempferol is from kaemperol-3-O-rutinoside, genkwanin is from swertisin, naringenin is from 5,7-Dihydroxy-2-(4-hydroxyphenyl)6,8-bis[3,4,5-trihydroxy-6(hydroxymethyl)oxan-2-yl]-2,3dihydrochromen-4-one, apigenin is from spinosin and its analogues, and isovitexin and vicenin-2, jujubogenin is from jujuboside A and jujuboside B.

### Anxiolytic Effect-Related Targets of Phytochemicals in SZJ Extract and Their Metabolites

In an integrated search of multiple databases, a total of 476 targets were found to be relevant with anxiety related disorders or diseases, of which 455 targets were acquired for 35 phytochemicals and 11 metabolites. All the interactions among the phytochemicals and targets are listed in **Supplementary Table 1**. After the two clusters were compared and analyzed, 71 target intersects were further determined and are listed in **Table 4**. Among the interactions of phytochemicals and metabolites on those 71 targets (data is not shown), (epi) catechin had the most interactions (degree =22), followed by kaempferol (degree =19), palmitic acid (degree =17), oleic acid (degree =15), betulinic acid (degree =14), apigenin (degree =14), zizyphusine (degree =12), and naringenin (degree =11), etc.

**Table 4:** Information of anxiolytic effects related targets of SZJ extract.

Gene Symbol	Full Name
ACSL4	acyl-CoA synthetase long-chain family member 4
ADIPOQ	adiponectin, C1Q and collagen domain containing
ADRA2A	adrenoceptor alpha 2A
AKR1C1	aldo-keto reductase family 1 member C1
AKT1	AKT serine/threonine kinase 1
ALDH2	aldehyde dehydrogenase 2 family (mitochondrial)
ALDH5A1	aldehyde dehydrogenase 5 family member A1
ANG	angiogenin
APP	amyloid beta precursor protein
ATP1A1	ATPase Na+/K+ transporting subunit alpha 1
ATP1A3	ATPase Na+/K+ transporting subunit alpha 3
BCAT2	branched chain amino acid transaminase 2
BDNF	brain derived neurotrophic factor
CAT	catalase
CCL3	C-C motif chemokine ligand 3
CHRNA7	cholinergic receptor nicotinic alpha 7 subunit
CNR1	cannabinoid receptor 1
CNR2	cannabinoid receptor 2
COMT	catechol-O-methyltransferase
COX1	cytochrome c oxidase subunit I
COX2	cytochrome c oxidase subunit II
CREB1	cAMP responsive element binding protein 1
CTNNB1	catenin beta 1
CXCL8	C-X-C motif chemokine ligand 8
CYP2E1	cytochrome P450 family 2 subfamily E member 1
DNMT1	DNA methyltransferase 1
DRD2	dopamine receptor D2
DRD3	dopamine receptor D3
DRD4	dopamine receptor D4
EDN1	endothelin 1
ESR1	estrogen receptor 1
ESR2	estrogen receptor 2
GABRA1	gamma-aminobutyric acid type A receptor alpha1 subunit
GABRA2	gamma-aminobutyric acid type A receptor alpha2 subunit
GABRA3	gamma-aminobutyric acid type A receptor alpha3 subunit
GABRA6	gamma-aminobutyric acid type A receptor alpha6 subunit
GABRG2	gamma-aminobutyric acid type A receptor gamma2 subunit
GLO1	glyoxalase I
GM2A	GM2 ganglioside activator
GPER1	G protein-coupled estrogen receptor 1
GRIN2A	glutamate ionotropic receptor NMDA type subunit 2A
GSK3B	glycogen synthase kinase 3 beta
GSTP1	glutathione S-transferase pi 1
HSD11B2	hydroxysteroid 11-beta dehydrogenase 2
HTR1A	5-hydroxytryptamine receptor 1A
HTR1B	5-hydroxytryptamine receptor 1B
HTR1D	5-hydroxytryptamine receptor 1D
HTR2A	5-hydroxytryptamine receptor 2A
HTR2B	5-hydroxytryptamine receptor 2B
HTR2C	5-hydroxytryptamine receptor 2C
HTR3A	5-hydroxytryptamine receptor 3A
ICAM1	intercellular adhesion molecule 1
IL6	interleukin 6
INS	insulin
MAPK1	mitogen-activated protein kinase 1
MIF	macrophage migration inhibitory factor (glycosylation-inhibiting factor)
ND1	NADH dehydrogenase, subunit 1 (complex I)
NOS2	nitric oxide synthase 2
NR1I2	nuclear receptor subfamily 1 group I member 2
NR3C1	nuclear receptor subfamily 3 group C member 1
PAH	phenylalanine hydroxylase
PON1	paraoxonase 1
SCN1A	sodium voltage-gated channel alpha subunit 1
SHBG	sex hormone binding globulin
SIRT1	sirtuin 1
SOD1	superoxide dismutase 1, soluble
SQSTM1	sequestosome 1
TGFBR2	transforming growth factor beta receptor 2
TNF	tumor necrosis factor
TP53	tumor protein p53
TRPV1	transient receptor potential cation channel subfamily V member 1

**Figure 2:**
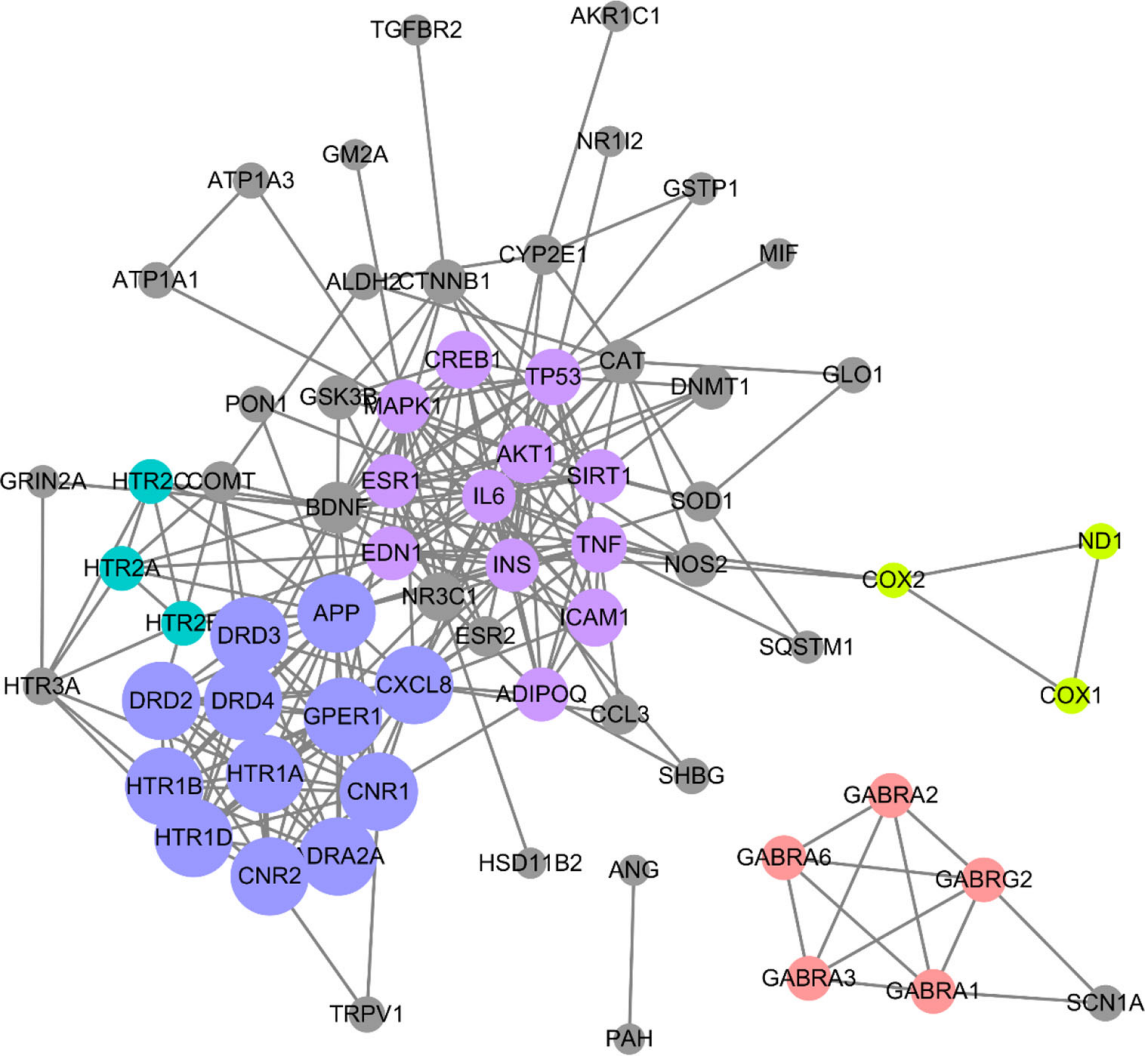
Protein-protein interaction (PPI) network of anxiolytic effects-related targets of SZJ. Cytoscape (Version 3.6.1) was applied to construct the interactions downloaded from the STRING (interaction score set as high confidence >0.7). All the targets are represented by nodes, whereas the interactions between the targets are represented by edges. MCODE plug-in was applied to conduct cluster analysis. Different clusters are noted with different colors. The node size is proportional to its located cluster MCODE score.

To screen out the core targets, PPI and MCODE cluster analysis was performed on 71 identified targets. As shown in **Figure 2**, 67 nodes plus 260 edges were obtained, in which the clustering coefficient is 0.564 and average number of neighbors is 7.761. With that, MCODE cluster analysis indicated 5 clusters. Specific data of target clusters were exported and are presented in **Table 5**. As a result, 35 core targets were obtained from these 5 clusters, suggesting the core anxiolytic effect targets of SZJ extract. Notably, most of these targets are neuroactive ligand receptors including serotonin (5-HT) receptors (e.g., HTR1A, HTR1B, HTR2A, HTR2B, HTR2C, and HTR1D), GABA_A_ receptors (e.g., GABRA1, GABRA2, GABRA3, GABRA6, and GABRG2), dopamine receptors (e.g., DRD2, DRD3, and DRD4), cannabinoid signaling (CNR1 and CNR2), and adrenergic (ADRA2A) and glutamate receptor (GRIN2A).

**Table 5:** List of genes clusters information analyzed by MOCDE on the base of PPI data downloaded from the STRING.

Cluster	Score (Density*#Nodes)	Nodes	Edges	Node IDs
1	10.154	12	66	GPER1, ADRA2A, DRD2, CNR2, CNR1, HTR1A, HTR1B, APP, DRD4, HTR1D, DRD3, CXCL8
2	8	12	52	CREB1, AKT1, TNF, SIRT1, TP53, MAPK1, EDN1, ICAM1, ADIPOQ, IL6, ESR1, INS
3	3.333	5	10	GABRA2, GABRG2, GABRA6, GABRA3, GABRA1
4	1.5	3	3	HTR2C, HTR2A, HTR2B
5	1.5	3	3	COX1, COX2, ND1

### GO and KEGG Pathway Enrichment and Analysis

**Figure 3:**
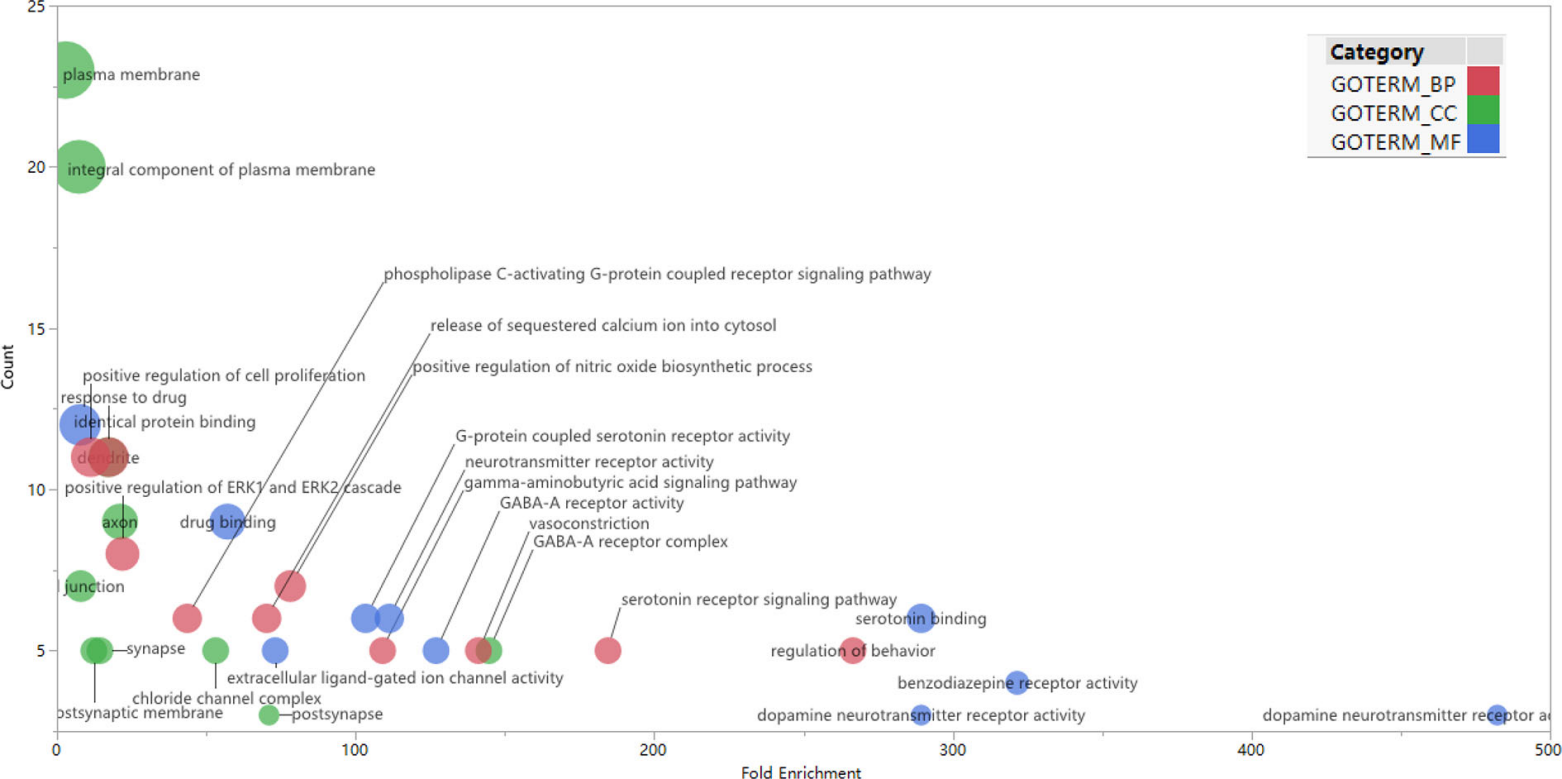
Top 10 significantly enriched GO terms in biologic process (red), cellular components (green), and molecular function (blue) categories. The bubble diagram was made using JMP software 14.2.0 (SAS Institute Inc., USA). The bubble size is proportional to its involved targets percentage in the term.

**Figure 4:**
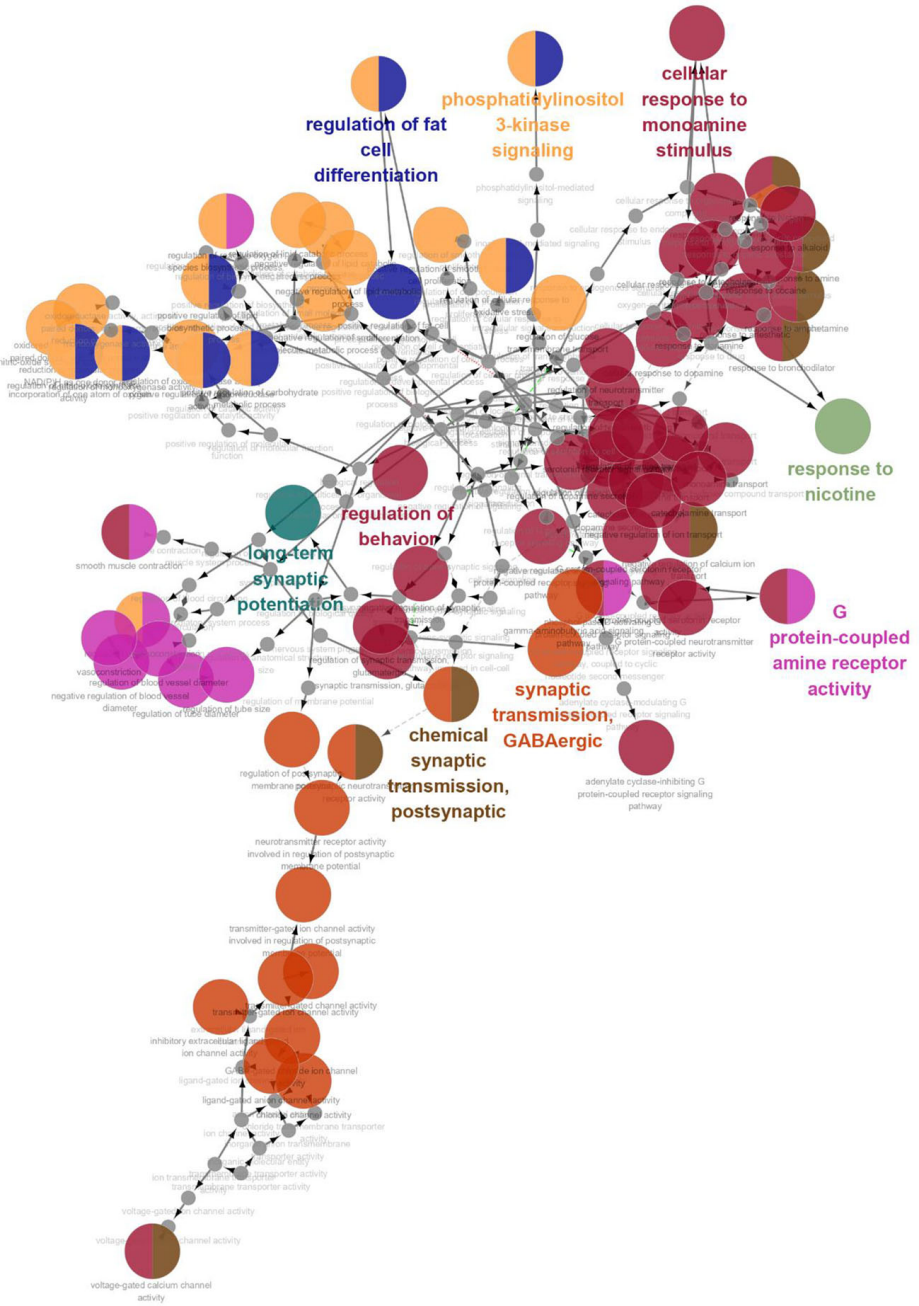
The interaction networks of enriched biological processes. ClueGO was applied to analysis procedure, and multiple color circles indicate that they are involved in multiple biological processes.

GO enrichment analysis was conducted on the 35 core targets by using DAVID. All the enriched GO terms are seen in **Supplementary Table 2**. The top 10 significant terms in biological process, molecular function, and cell component categories are shown in **Figure 3**. The results demonstrated that GO terms were mainly concentrated in neurotransmitter receptors signaling, particularly GABA and serotonin receptor signaling. Functions of activating neurotransmitter receptors and ligand-gated ion channel *via* receptor complex, and regulating synaptic transmission were mainly involved. Cytoscape ClueGo plug-in was further applied to visualize the interaction network of biological process, as shown in **Figure 4**. All statistically significant biological processes were listed in **Supplementary Table 3**.

**Figure 5:**
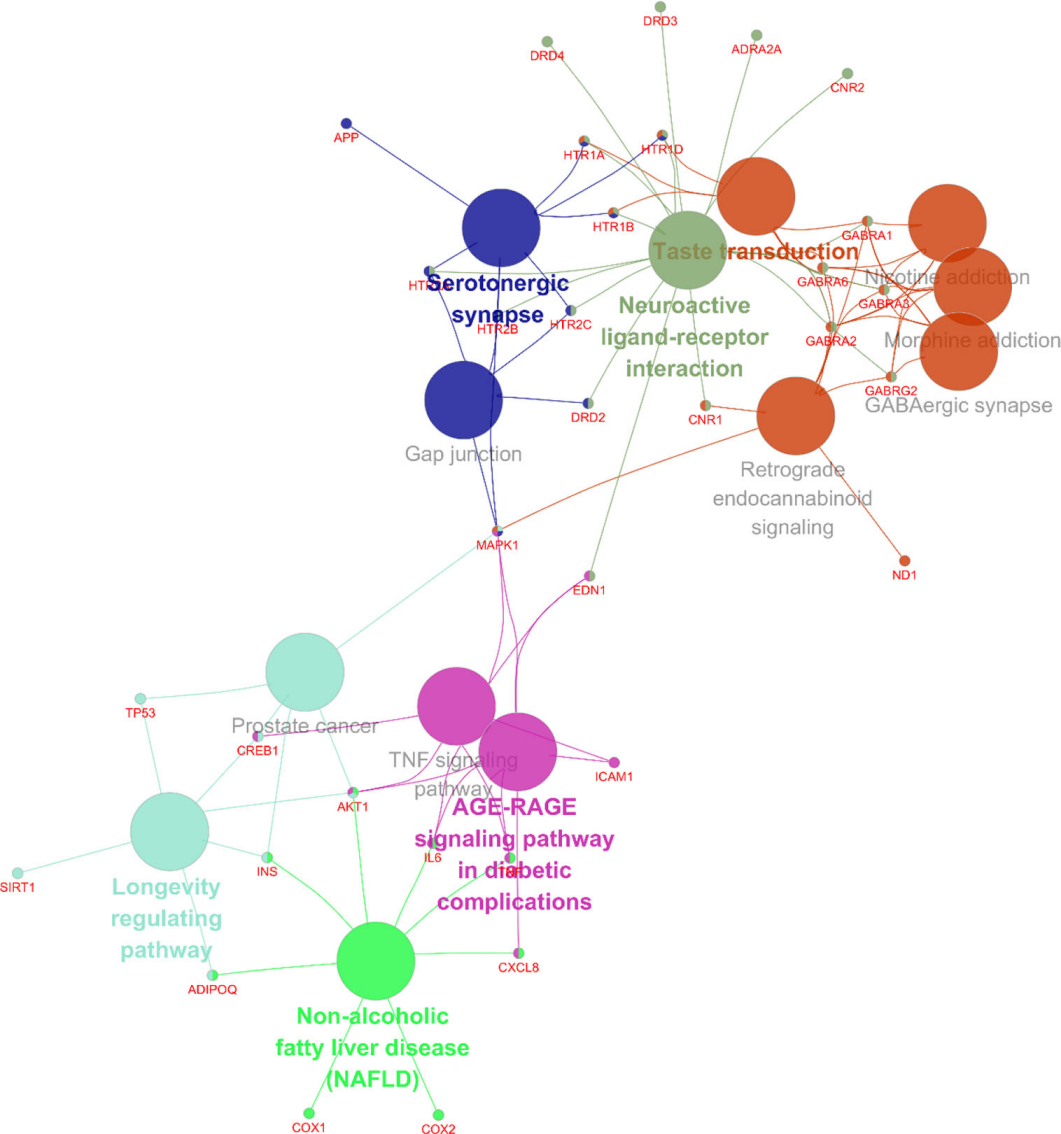
Targets-pathway network associated with anxiolytic effects of SZJ extract. A cytoscape ClueGo plug-in was applied to enrich the pathways and construct the network.

In addition, 35 identified core targets were imported to ClueGo for KEGG pathway enrichment, resulting in 14 statistically significant pathways. The targets-pathway network is shown in **Figure 5**, demonstrating that neuroactive ligand-receptor interaction is the most significant pathway with involvement of 18 targets, followed by serotonergic synapse pathway (8 targets), taste transduction pathway (7 targets), etc. Other nervous system related pathways including GABAergic synapse and retrograde endocannabinoid signaling, signaling transduction related pathways including TNF signaling pathway, and longevity regulating pathways were also significantly enriched. These results are consistent with the results from GO enrichment analysis. Taken together, these findings suggest that SZJ extract mainly exerts an anxiolytic effect *via* modulation of serotonergic and GABAergic systems.

GABA_A_ receptors in the central nervous system are known as the major targets for the first line treatment of anxiety (Poisbeau et al., 2018). GABA_A_ receptors subunits, mainly including GABRA1, GABRA2, and GABRA3, have been reported to mediates anxiolysis (Möhler, 2012). Similarly, preclinical studies have suggested that most 5-HT receptors subtypes participate in anxiety-like processes, and blocking or stimulating individual 5-HT receptor subtypes might cause the anxiolytic-like effect (Żmudzka et al., 2018). HTR1A, HTR1B, HTR2A, and HTR2B were commonly targeted in preclinical studies for the anxiety treatment (Graeff et al., 1996; Clinard et al., 2015; Spiacci et al., 2016; Colangeli et al., 2019) as well as the anxiolytic-like studies of SZJ (Wang et al., 2010; Liu et al., 2015). Hence, among the potential targets acted by SZJ, GABRA1, GABRA2, GABRA3, HTR1A, HTR1B, HTR2A, and HTR2B were preferentially selected for further validation.

### Effects of SZJ Extract on mRNA Expression of GABA_A_ and 5-HT Receptor Subtypes

**Figure 6:**
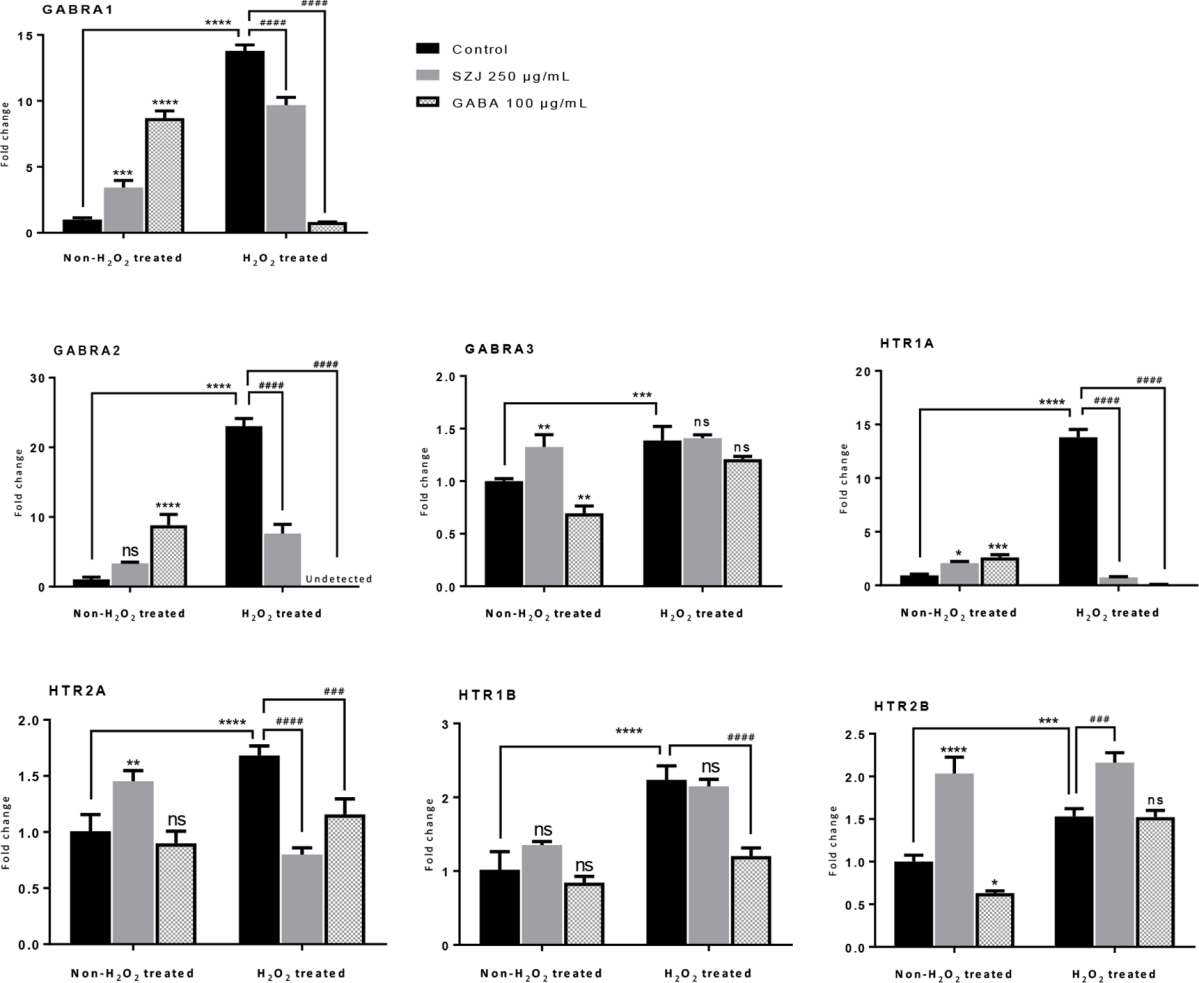
Effects of SZJ extract on mRNA gene expressions of different subtypes of GABA_A_ and 5-HT receptors. GraphPad Prism 7.0 software was applied for statistical analysis and graphing. All data were expressed as mean ± SD, and a two-way ANOVA followed by Tukey’s test was applied. Two-way ANOVA analysis results presented that, GABRA1, F (2, 12) =57.27: p < 0.0001; GABRA2, F (2, 12) =112.50: p < 0.0001; GABRA3, F (2, 12) =40.10: p < 0.0001; HTR1A, F (2, 12) =677.80: p < 0.0001; HTR2A, F (2, 12) =13.07: p = 0.0010; HTR1B, F (2, 12) =42.99: p < 0.0001; HTR2B, F (2, 12) =151.50: p < 0.0001. Post hoc Tukey’s test results were represented by comparing with non-H_2_O_2_ treated control group, ^*^p < 0.05, ^**^p < 0.01, ^***^p < 0.001 and ^****^p < 0.0001; compared with H_2_O_2_ treated control group, ^###^p < 0.001 and ^####^p < 0.0001.

Based on the findings from system biology analysis, RT-qPCR was employed to evaluate the effects of SZJ extract on mRNA expression of GABRA1, GABRA2, GABRA3, HTR1A, HTR1B, HTR2A, and HTR2B. As shown in **Figure 6**, in the non-H_2_O_2_ treated cells, GABA 100 μg/mL significantly enhanced the expression of GABRA1 (p< 0.0001), GABRA2 (p< 0.0001), and HTR1A (p< 0.001) comparing with the control group, while it significantly decreased the expression of GABRA3 (p< 0.01) and HTR2B (p< 0.05). Similar as GABA, SZJ extract 250 μg/mL exhibited significant effect on enhancing expression of GABRA1 (p< 0.001) and HTR1A (p< 0.05). However, contrast to GABA group, obvious expression enhancement of GABRA3 (p< 0.01) and HTR2B (p< 0.0001) were observed at 250 μg/mL SZJ extract. These differences in regulating GABA_A_ receptor subtypes following SZJ and GABA stimulations could help to explain the different mechanisms of anxiolytic effect of the two ingredients.

Furthermore, because oxidative stress mechanisms have been well explored in anxiety disorders (Bouayed et al., 2009; Salim, 2017), the effects of SZJ extract on GABA_A_ and 5-HT receptors were also evaluated in hydrogen peroxide (H_2_O_2_) induced oxidative stress condition. As a result, a remarkable increase (p< 0.0001) in GABRA1, GABRA2, HTR1A, HTR1B, and HTR2A expression following 100 μM H_2_O_2_ stimulation was observed. Currently, though the alteration of GABA_A_ and 5-HT receptors under strong oxidative stimulation is unclear, our result in some extent suggested that overexpression of GABRA1, GABRA2, HTR1A, HTR1B, and HTR2A may be involved in oxidative stress-induced anxiety. Intriguingly, the sharp increase induced by H_2_O_2_ in GABRA1, GABRA2, HTR1A, and HTR2A were significantly antagonized by both SZJ extract 250 μg/mL and GABA 100 μg/mL. Hence, inhibition of GABRA1, GABRA2, HTR1A, and HTR2A overexpression is also possibly involved in anxiolytic-like mechanisms of SZJ.

## Discussion

The identification of phytochemicals in herbal materials is a critical step during the process of system biology analysis. Herbal materials are often subjected to extraction, concentration, and/or purification, resulting in the phytochemical compositions alteration. The phytochemical data from current databases (e.g., TCMSP, http://tcmspw.com/tcmsp.php; TCMID, http://www.megabionet.org/tcmid/) may not be used directly for system biology investigation. Additional methods for phytochemical identification, such as UPLC-Q-TOF/MS, should be a complementary tool to obtain more accurate results of phytochemical compositions (Shen et al., 2013). In current work, a series of flavonoid glycosides and saponins were identified from SZJ extract, in which the spinosin derivatives including 6’’’-vanilloylspinosin, 6’’’-para-hydroxylbenzoylspinosin, 6’’’-sinapoylspinosin, 6’’’-para-coumaroylspinosin, 6’’’-feruloyspinosin, 6’’’-(-)-phaseoylspinosin, and 6’’-O-feruloylspinosin are rare in other plant species. The studies on their bioactivities and effective targets have been so poorly reported that there is not enough data for system biology analysis. In addition, their chemical structures are complicated and contain multi-chiral centers that bring a great challenge to obtain the potential targets through reverse virtual fishing technique. Traditional system biology analysis would take ADME (absorption, distribution, metabolism, and excretion) screening strategy that may exclude these glycosides with low oral bioavailability and low drug-likeness (Yue et al., 2017a; Yue et al., 2017b). For instance, ginsenosides are the dominant phytochemicals in ginseng and are thought to be contributed to its multiple bioactivities (Ru et al., 2015; Kim et al., 2017). However, the above analysis strategy, i.e., ADME screening, would exclude the ginsenosides along with their contributions on efficacy when performing systematic research of ginseng. Therefore, such an analytical strategy is incomplete and not systematic. In fact, it has been well demonstrated that the metabolites of the ginsenosides are responsible for the specific bioactivities (Chen et al., 2018; Kim, 2018). Similarly, the chemoinformatics and pharmacoinformatics approach indicated that jujubogenin was the effective GABA_A_ agonist, neither jujuboside A nor jujuboside B (Chen, 2009). Gut microbes play an important role in favoring phytochemicals transformation into metabolites endowed with biological activity (Dey, 2019). As a result, the strategy that involves the metabolites of glycosides in gastrointestinal environment (e.g., gut microbes) will be a more reasonable approach to understand the actual efficacy and mechanism of herbal materials in which the glycosides are considered as the main active components.

The GABA_A_ receptors are chloride channels and are composed of several subunit classes (α, β, γ, δ, and ϵ) (Olsen and Sieghart, 2008). GABAergic neurotransmission plays an important role in anxiety status. Previous studies have shown that deficit of GABA_A_ receptors and reduction of GABA transmission were observed in people with anxiety-like symptoms (Horowski and Dorow, 2002; Nutt and Malizia, 2004; Hasler et al., 2008). In contrast, positive modulation of GABA_A_ receptors and enhancement of GABA transmission have shown anxiolytic effects. Classic benzodiazepines reduce anxiety by interacting with the GABA_A_ receptors *via* the benzodiazepine binding site, which is present at the interface of α1, α2, α3, or α5 subunits and γ subunit of GABA_A_ receptors (Möhler, 2012). Other classes of compounds, GABA, barbiturates, and alcohol also could act at different benzodiazepine binding sites to increase the opening of the chloride channel resulting in enhancement of inhibitory synaptic transmission (Harris, 1990; Schousboe and Redburn, 1995). The results of our system biology study suggested the GABA_A_ receptors signaling is a significant pathway involving in anxiolytic effect of SZJ. In fact, pharmacologic study has found that spinosin, a major C-glycoside flavonoid in SZJ, exerted anxiolytic-like effects *via* modulation of GABA_A_ and 5-HT receptors (Liu et al., 2015). Similarly, 6′′′-feruloylspinosin and spinosin have been reported to significantly enhance the expression of GABRA1 and GABRA5 mRNA in rat hippocampal neurons (Qiao et al., 2016). In addition, it has been found that stimulation of jujuboside A at 50 µg/mL could increase the mRNA transcription levels of GABRA1, GABRA5, GABRB1, and GABRB2 in hippocampal neurons (You et al., 2010; Wang et al., 2015); however, long time stimulation of jujuboside A at a high dose of 100 µg/mL result in the decrease of GABRA1 and GABRB2 mRNAs expression (You et al., 2010). These results suggested a two-way modulatory effect of SZJ on GABRA1 and other GABA_A_ receptors. Such benefits were similar to what we found in this work, that is, SZJ extract enhanced mRNA level of GABRA1 in non-H_2_O_2_ treated SH-SY5Y cells, but inhibited the H_2_O_2_-induced overexpression of GABRA1. Therefore, combining with the results from literatures and our results, it was suggested that SZJ exhibited anxiolytic effects through modulating GABA_A_ receptors, in which a two-way modulation of GABRA1 may play an important role.

It was well established that the alteration of various behaviors in anxiety disorders including appetite, mood, sleep, and cognitive function have been linked to the serotonergic system (Liu Y. et al., 2018; Liu et al., 2019). Serotonin receptors are prevalent throughout the nervous system and the periphery, and they potentially control the serotonergic neurotransmission throughout the brain and neuronal activity to alleviate neuropsychiatric disorders (Okazawa et al., 1999). Generally, the activation of HTR1A, HTR2A receptors can produce anxiolytic effects, whereas inactivation of them increases anxiety-like behaviors (Clinard et al., 2015; Spiacci et al., 2016). Involvement of other 5-HT receptors including HTR1B, HTR1B, and HTR2C in the mechanisms of anxiety have also been recognized (Graeff et al., 1996; Griebel et al., 1997; McCorvy and Roth, 2015). Similarly, our system biology analysis found that the serotonergic synapse pathway was dominant in anxiolytic effects mechanism of SZJ extract, in which different subtypes of 5-HT receptors were involved. Notably, as the same effect on GABRA1, SZJ extract also showed a two-way modulation on HTR1A and HTR2A in our RT-qPCR test. Genetic studies in animal models have suggested that anxiety-like behavior can increase when the HTR1A function is eliminated or overexpressed (Overstreet et al., 2003). Hence, these results suggested the involvement of modulating serotonergic synapse pathway, specifically two-way modulation of HTR1A and HTR2A in anxiolytic effects of SZJ.

In addition, the cannabinoid receptors (CNR) are extensively expressed in areas of the nervous system and have been found closely associated with anxiety behavior (Akirav, 2011). It has been well illustrated that endocannabinoid (eCB) reduces the serotonin release in the central nervous system and increases the expression and function of HTR1A in the hippocampus *via* the activation of CNR1 (Haj-Dahmane and Shen, 2011; Patel et al., 2017). Beyond CNR1, eCB system could exert actions on other targets including CNR2, transient receptor potential vanilloid receptor type 1 (TRPV1), or cyclooxygenase-2 (COX2) to participate in improvement of anxiety (Patel et al., 2017). In addition, cyclic AMP-responsive element-binding protein (CREB) has been suggested to be crucial for the role of HTR1A in modulating anxiety-related behaviors *via* mediating hippoacampus structural plasticity (Zhang J. et al., 2016). Intriguingly, this systematic analysis work showed phytochemicals in SZJ extracts potentially act on the above mentioned targets including CNR1, CNR2, TRPV1, COX2, and CREB. These results, to some extent, suggested that the mechanism of action of SZJ in anti-anxiety may also involve those pathways/targets that indirectly modulate eCB and serotoninergic systems. More attention needs to be paid to those targets/pathways in further experimental studies on anxiolytic effects of SZJ.

**Figure 7:**
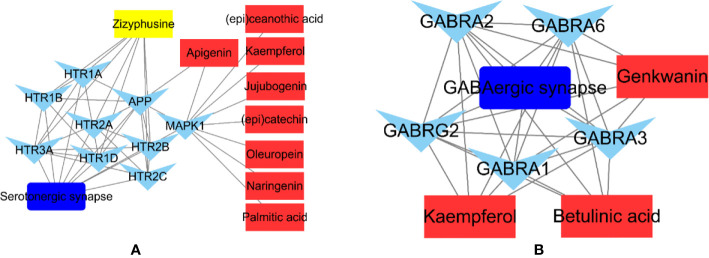
Abstracted phytochemicals-targets-pathway sub-networks of serotoninergic synapse **(A)** and GABAergic pathways **(B)**. Cytoscape (Version 3.6.1) was applied to construct the sub-networks.

The phytochemicals in herbal medicines are the substantial basis for their pharmacologic actions. Those phytochemicals with good bioactivity and high content are considered to be the chemical markers in quality control of the herbal medicines. Jujuboside A and spinosin are used to quality markers of SZJ crude drug in Chinese Pharmacopoeia (Edition 2020). Combining the results from literatures reports (Han et al., 2009; Abdoul-Azize, 2016) and our results, the modulations of GABAergic and serotoninergic systems seem the major mechanisms of SZJ exerting anxiolytic effects, as well as the traditional efficacy of nourishing heart and calming mind. Based on that, we abstracted the phytochemicals-targets-pathway sub-networks of GABAergic and serotoninergic synapse pathways. As shown in **Figure 7**, it demonstrated that metabolites of C-glycosides (spinosin, etc.) and jujubosides (jujuboside A, etc.) including apigenin, kaempferol, naringenin, genkwanin, and jujubogenin were involved in modulation of GABAergic and serotoninergic synapse pathways. The result provides the extra evidence to support that C-glycosides and jujubosides are responsible for the anxiolytic effects of SZJ, and they support jujuboside A and spinosin as chemical markers for quality control of SZJ and its preparations. Beyond the C-glycosides and jujubosides, the involvements of triterpenic acid (e.g., betulinic acid) and alkaloid (zizyphusine) were also observed in modulation of GABAergic and serotoninergic synapse pathways. Specifically, betulinic acid is a function of the modulating GABAergic system *via* multiple subtypes of GABA_A_ receptors, whereas zizyphusine is a function of the modulating serotoninergic system *via* multiple subtypes of 5-HT receptors. Notably, it has been reported that zizyphusine was identified as one of the principal components in SZJ by principal component analysis (Sun et al., 2014). And according to records in TCMIP, zizyphusine is exclusively derived from *Ziziphus jujube* (fruit or seeds), and its bioavailability and druglikeness is much better than C-glycosides and jujubosides. These findings recommend that the involvement of zizyphusine in quality control of SZJ extract and pharmacologic actions in anxiolytic effect is worth investigating in the future. Because the pharmacologic study of zizyphusine is poor at present, more attention could be paid to research and development of zizyphusine as a potential natural anti-anxiety drug.

There are some limitations of our current research. First, we did not conduct the behavioral test to confirm the anxiolytic-like effect of SZJ. Such benefit of SZJ was concluded on the base of previous pharmacodynamic studies, as well as clinical practice experience of traditional medicine. Our *in vitro* mRNA expression evaluation of GABA_A_ and 5-HT receptors only used a single concentration of SZJ extract, which corresponds to approximately 90% cell viability in CellTiter-Glo assay. Leveraging the integrated approach of system biology, UPLC-Q-TOF/MS and RT-qPCR, present work contributed to the illustration of potential mechanism of action involved in the anxiolytic-like effect of SZJ. However, further in-depth preclinical studies are warranted to verify the results obtained from the current analysis.

## Conclusions

In conclusion, our results systematically demonstrated that anxiolytic mechanisms of SZJ mainly involved the regulation of serotonergic and GABAergic synapse pathways, in which the two-way modulation of GABRA1, HTR1A, and HTR2A may play an important role. In addition to C-glycosides and jujubosides, triterpenic acids and zizyphusine identified in SZJ also contributed to the regulation of serotonergic and GABAergic synapse pathways. This study provides directional predictions of anxiolytic mechanism of SZJ and insights to improve the quality control of standard extraction.

## Data Availability Statement

The raw data supporting the conclusions of this article will be made available by the authors, without undue reservation, to any qualified researcher.

## Author Contributions

LC, XZ, and JK designed the study. XZ, YZ, LZ, and BL performed the experiments. LC, CH, and JD prepared the manuscript. All authors contributed to the article and approved the submitted version.

## Funding

The Nutrilite Health Institute fully funded this study.

## Conflict of Interest

The authors declare that the research was conducted in the absence of any commercial or financial relationships that could be construed as a potential conflict of interest.

## Supplementary Material

Supplementary Table 1 Acquired targets for identified phytochemicals in SZJ extract.

Supplementary Table 2 Enrichment results of GO terms including biological process, cellular component, and molecular function. DAVID bioinformatics platform (version 6.8) was used for analysis.

Supplementary Table 3 The information of enriched biological processes.
